# Therapeutic Potential of Hydrogen as a Radioprotective Agent for the Prevention of Radiation Dermatitis

**DOI:** 10.3390/antiox13121475

**Published:** 2024-11-29

**Authors:** Deng-Yu Kuo, Yu-Chi Wang, Pei-Han Chou, Chen-Wei Lai, Fu-I Tung, Tse-Ying Liu

**Affiliations:** 1 Division of Radiation Oncology, Department of Radiology, Far Eastern Memorial Hospital, New Taipei 220, Taiwan; dykuo.be12@nycu.edu.tw; 2 Department of Biomedical Engineering, National Yang Ming Chiao Tung University, Taipei 112304, Taiwan; tomato13590.be04@nycu.edu.tw (Y.-C.W.); peihan941@gmail.com (P.-H.C.); a.wei.be10@nycu.edu.tw (C.-W.L.); 3 Department of Orthopaedics, Yang-Ming Branch, Taipei City Hospital, Taipei 111024, Taiwan; dag47@tpech.gov.tw; 4 Department of Health and Welfare, College of City Management, University of Taipei, Taipei 111036, Taiwan

**Keywords:** hydrogen gas, radiation dermatitis, antioxidant, anti-inflammatory, anti-apoptosis

## Abstract

Radiation dermatitis (RD) is a common side effect in patients receiving radiotherapy. Currently, clinical skincare approaches for acute RD vary widely among institutions and lack consensus. Hydrogen molecules, acting as radioprotective agents by selectively scavenging free radicals, have the potential to protect against RD. In this study, we demonstrate that hydrogen reduces double-strand breaks, mitochondrial depolarization, and inflammatory cytokines induced by irradiation damage in HaCaT cells. Furthermore, in vivo experiments reveal that exposing irradiated skin areas to a hydrogen gas environment alleviates RD. Assessment of skin appearance grade and histology staining revealed that direct transdermal application of hydrogen can prevent radiation-induced follicle damage, dermal thickening, and leukocyte infiltration, thereby reducing the severity of RD. In addition, hydrogen enhances the skin’s antioxidant capacity, leading to a reduction in the Bcl-2-associated X protein/B-cell lymphoma 2 (Bax/Bcl-2) ratio, the number of apoptotic cells, and the expression of pro-inflammatory cytokines. Our data demonstrate that hydrogen possesses antioxidant, anti-inflammatory, and anti-apoptotic properties, and could be a preventive strategy for RD.

## 1. Introduction

Radiation therapy (RT) is one of the treatment modalities for cancer, which is applied to approximately 60% of cancer patients [1]. Ionizing radiation can produce free electrons and free radicals, triggering an inflammatory response and oxidative stress. These processes interact, mutually reinforcing each other, ultimately causing cell damage and death [2,3]. When radiation kills cancer cells, it can also damage the surrounding normal tissues. A prevalent side effect of RT is acute radiation dermatitis (RD), which occurs in up to 95% of patients undergoing treatment. RD affects patients’ appearance and quality of life, subsequently diminishing their willingness to continue with the treatment [4]. The pathogenesis of RD involves direct radiation injury and subsequent inflammation in the epidermis, dermis, and vasculature. Acute reactions cause changes in skin pigmentation, interrupted hair growth, and dermal damage. Higher doses lead to increased mitosis in basal keratinocytes, resulting in thickened, scaly skin (dry desquamation). At even higher doses, the basal layer cannot recover, causing moist desquamation. These damages compromise the skin’s barrier and immune function, increasing the risk of infection. Additionally, vascular endothelium damage leads to hypoxia and increases the expression of transforming growth factor (TGF)-β. TGF-β then stimulates fibroblasts to produce excessive extracellular matrix (ECM) proteins, resulting in fibrosis [5,6].

Based on the mechanisms of radiation-induced damage, radiation protection methods can be broadly categorized into four types: promoting DNA damage repair, reactive oxygen species (ROS) scavenging, anti-inflammation, and inhibition of death signaling pathways [7]. However, clinical skincare approaches for acute RD vary widely among institutions, relying heavily on the expertise and opinions of individual clinicians. Recently, the Multinational Association of Supportive Care in Cancer (MASCC) Oncodermatology Study Group has developed clinical guidelines based on a systematic review summarizing existing evidence on the management of acute RD. These guidelines recommend various topical products, including corticosteroids like mometasone and betamethasone, olive oil, and polyurethane barrier films [4,8]. While topical steroids have shown efficacy in preventing RD in patients undergoing RT for head and neck or breast cancer [9,10], they can cause side effects such as skin atrophy and increased infection risk. Olive oil, known for its antioxidant and anti-inflammatory properties due to its oleic acid content [11], may leave a greasy feeling on the skin and make maintaining clear RT markings challenging. Barrier films moisturize and protect the skin, reducing the risk of external mechanical injuries or bacterial infections and thus decreasing RD occurrence [12]. However, it is essential to consider that applying topical agents or a barrier film may increase the skin dose in photon RT due to the bolus effect associated with their thickness [13].

Hydrogen, with a low molecular weight, can easily penetrate biological membranes and diffuse into the cell nucleus and mitochondria. The delivery of hydrogen can be achieved through various routes, including inhalation, oral intake of hydrogen-rich water, injection of hydrogen-rich saline, increased intestinal hydrogen, and direct incorporation [14]. Hydrogen shows promise as a radioprotective agent by selectively scavenging hydroxyl radicals (•OH) and peroxynitrite anions (ONOO−), potent ROS [15,16]. The proven antioxidant, anti-inflammatory, and anti-apoptotic properties of hydrogen render it a therapeutic gas suitable for various conditions. These include ischemic organ injuries, neurodegenerative disorders, bone and joint ailments, and respiratory diseases [16,17]. Hydrogen shows potential as a valuable treatment option for preventing and managing skin-related illness, with its application increasingly seen in clinical settings for patients [18,19]. Additionally, the skin, located on the body surface, is highly amenable to direct incorporation of hydrogen, whether through bathing or gas exposure, without leading to the accumulation of doses in the body. Nevertheless, there is currently limited literature specifically focusing on the protective effects of molecular hydrogen on RD [20,21,22]. Existing studies have not thoroughly explored the protective mechanisms of hydrogen against RD, and the routes employed may not precisely regulate the delivery of hydrogen to the skin.

In this study, we simulated the mechanism of RD through in vitro tests and verified the hypothesis of the protective effects of hydrogen. In animal experiments, a chamber was designed to expose the mouse’s skin to a high concentration of hydrogen gas, anticipating enhanced hydrogen penetration into the skin. The goal is to achieve preventive effects against RD and explore the regulation of oxidative, inflammatory, and apoptotic-related molecules in the skin.

## 2. Materials and Methods

### 2.1. In Vitro Test

#### 2.1.1. Cell Culture

The human epidermal keratinocyte HaCaT (T0020001, AddexBio, San Diego, CA, USA) cell line was used. Cells were cultured in DMEM medium containing 10% fetal bovine serum (FBS) and 1% penicillin/streptomycin at 37 °C and 5% CO_2_. Cells were passaged every two days. Cultivation was performed in 10 cm dishes (BIOFIL JET-TCD-101, Guangzhou, China).

#### 2.1.2. Radiation Delivery

For cell exposure, a dose of 4 Gy was delivered in a single session with cells positioned 5 cm from the source. The X-ray machine used was the Nanoray (NS-082505, NanoRay Biotech Co., Ltd, Taipei, Taiwan), equipped with an X-ray source Nanoray (NS-08P-01, NanoRay Biotech Co., Ltd, Taipei, Taiwan), operating at an energy of 50 keV and a dose rate of 3.88 Gy/min (Figure S1).

#### 2.1.3. Double-Strand Break Assay

HaCaT cells were seeded in a 12-well plate (Corning^®^ Costar^®^, Corning Incorporated, Corning, NY, USA) with an 18 mm cover glass (MARIENFIED, Lauda-Königshofen, Germany) at a density of 8 × 10^4^ cells per well. Cells were cultured at 37 °C and 5% CO_2_ for 24 h. Samples designated for hydrogen treatment were transferred to a hydrogen supply chamber for 1 h of exposure. Following hydrogen exposure, the XR groups underwent X-ray irradiation at room temperature. After a 30 min incubation at 37 °C, cells were fixed with 4% formaldehyde in double-distilled water (DDIW, Merck Millipore, Darmstadt, Germany) at room temperature for 10 min, permeabilized with 0.1% Triton X-100 in DDIW for 10 min at room temperature, and then blocked with 2.5% FBS in Tris-buffered saline with Tween 20 (TBST) at 4 °C for 1 h. Cells were then incubated with primary antibodies (1:500, Histone H2A.X S139ph, GTX127340, GeneTex, San Antonio, TX, USA) at 4 °C overnight, followed by secondary antibody incubation (1:1000, Goat Anti-Rabbit IgG, DyLight 488, GTX213110-04, GeneTex, San Antonio, TX, USA) at room temperature for 1 h. Finally, Fluoroshield™ with 4′,6-diamidino-2-phenylindole (DAPI, GTX30920, GeneTex, San Antonio, TX, USA) at a concentration of 1.5 μg/mL was applied to the coverslip, which was mounted onto a glass slide, and cells were imaged using a fluorescent microscope (Leica 6000B, Leica, Wetzlar, Germany).

#### 2.1.4. Mitochondrial Membrane Potential Detection

After treating 5 × 10^4^ HaCaT cells with hydrogen gas for 1 h, the cells were immediately exposed to radiation. A positive control group was treated with 10 µmol/L Carbonyl cyanide 4-(trifluoromethoxy) phenylhydrazone (FCCP, Cat. No. SML2959, Sigma Aldrich, St. Louis, MO, USA), which disrupts the mitochondrial membrane potential (MMP) by uncoupling the proton gradient, resulting in green fluorescence in JC-1-stained cells. Following irradiation, the cells were incubated for 3 h at 37 °C, and then collected using 0.25% trypsin for 3–5 min and centrifuged to remove the culture medium. The cells were resuspended in PBS containing JC-1 dye (Cat. No. 30001, BIOTI-UM, Fremont, CA, USA) for 15 min at 37 °C to evaluate the MMP. Flow cytometry (Beckman CytoFLEX, Brea, CA, USA) was then performed immediately. The gating strategy included (1) FSC-A vs. SSC-A to exclude debris and select the main cell population and (2) FSC-H vs. FSC-A to remove aggregates and ensure single-cell analysis. Fluorescence analysis was conducted using the PE channel for red fluorescence and the FITC channel for green fluorescence. A red/green fluorescence dot plot was generated to distinguish cells based on MMP. Healthy cells with a high MMP displayed strong red fluorescence, while cells with a reduced MMP emitted green fluorescence. FCCP-treated cells served as a reference for data interpretation.

#### 2.1.5. Pro-Inflammatory Cytokines

HaCaT cells (6 × 10^4^) were seeded in 24-well plates (Costar^®^, COS3524, Corning Incorporated, New York, NY, USA) and allowed to incubate for 24 h until cell adhesion occurred. The plates were then placed into a hydrogen gas supply chamber, where hydrogen gas was administered for 1 h at a flow rate of 1.2 L/min. Immediately following the hydrogen treatment, the cells were exposed to a radiation dose of 4 Gy. Twenty-four hours after irradiation, the supernatant from the cell cultures was collected. The collected supernatant was subjected to centrifugation at 13,200 rpm for 15 min at 4 °C to remove cellular debris and fragments. Subsequently, the clear supernatant was extracted and the interleukin-6 (IL-6) content in the culture medium was quantified using an ELISA kit (BioLegend, Cat. No. 430501, San Diego, CA, USA).

### 2.2. Ex Vivo Test

#### Hydrogen Transdermal Test

Three microliters (3 µL) of the MB-Pt probe (H_2_ Sciences Inc., Plano, TX, USA), a methylene blue probe that reacts with hydrogen to produce a colorless solution, was added to 0.6 mL of deionized water (DIW), and the hydrogen detection reagent was prepared. This reagent was then transferred to a 1.5 mL Eppendorf tube, onto which fresh skin samples from 6- to 8-week-old female BALB/c mice were placed, ensuring alignment with the gas outlet of the hydrogen gas chamber. The skin was affixed to the opening of the Eppendorf tube using tissue glue and secured with tape. A hydrogen delivery tube was then positioned to direct hydrogen gas (at a flow rate of 1.2 L/min) onto the skin surface. Reagent samples were collected from the microcentrifuge tubes at time points 0, 1, 2, and 3 h. This probe transitions from blue to transparent (colorless) when exposed to hydrogen. The absorbance of the reagent was measured at 662 nm using a Tecan Infinite 200 plate reader (TECAN Sunrise, Infinite 200 Pro, Morgan Hill, CA, USA).

### 2.3. In Vivo Test

#### 2.3.1. Animals

BALB/c female mice aged 6–8 weeks were obtained from BioLASCO Taiwan Co., Ltd, Nangang, Taipei, Taiwan. The mice were housed under controlled conditions, with a temperature range of 20–26 °C, humidity between 30 and 70%, and a 12 h light–dark cycle. They were provided with a sterilized lab diet. All animals used in our experiments were treated and housed according to a protocol approved by the Institutional Animal Care and Use Committee of National Yang Ming Chiao Tung University (IACUC-NYCU 1110320r).

#### 2.3.2. Radiation Dermatitis Model

Two days before the experiment, the hair on the back of the mice was removed using a hair removal cream (Nair Cream; active ingredient: sodium hydroxide) over an area of 1.5 × 1.5 cm². The mice were anesthetized by intraperitoneal injection of Ketamine (10 mg/100 g) and Xylazine (1.5 mg/100 g). The back skin of the mice was secured with bamboo sticks and rubber bands to maintain a flat position on the stage, while the remaining body parts were shielded with a lead plate (Figure S1). The exposed skin area received a cumulative dose of 35 Gy, delivered as 7 Gy per day over five consecutive days. Mice were anesthetized for each irradiation fraction to ensure stability and accuracy of exposure. The same machine model was used for both cells and animals. However, due to differences in exposure fields, animals were positioned 12 cm from the source, with a dose rate of 0.68 Gy/min.

#### 2.3.3. Design of Hydrogen Supply Chamber

The hydrogen supply chamber was divided into two compartments: one for anesthesia administration and the other for hydrogen exposure (Figure S2). A neck plate served as a partition to secure the mice at the neck, with their heads positioned in the anesthesia chamber and their bodies in the hydrogen chamber (Figure S3).

#### 2.3.4. Hydrogen Gas Treatment

The mice were anesthetized with isoflurane and positioned within a specialized chamber. Eye ointment (Betasom-N) was applied to prevent dryness. Their necks were secured with a neck plate, with their heads placed in the gas anesthesia compartment and their bodies in the hydrogen supply compartment, ensuring minimal gas mixing between the two sides. The gas anesthesia chamber was supplied with a mixture of oxygen and isoflurane, while the hydrogen supply chamber received hydrogen gas at a flow rate of 1.2 L/min. The back skin of the mice was exposed to hydrogen gas for approximately 2 h during each treatment fraction.

#### 2.3.5. Experimental Design

**Scheme 1:**
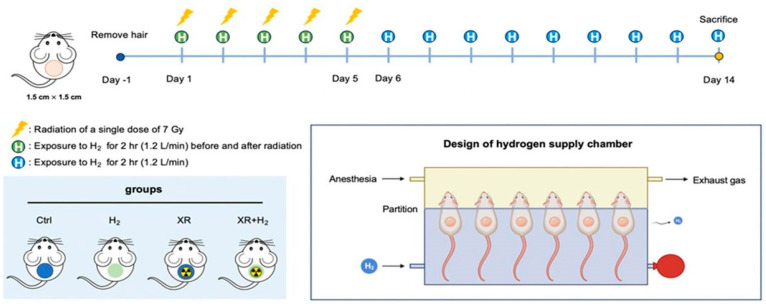
Experimental design of hydrogen therapy in BALB/c mice exposed to X-ray irradiation.

The detailed experimental design is shown in Scheme 1. BALB/c mice were randomly distributed into four groups: the Ctrl group (room air and sham-irradiated), the H_2_ group (hydrogen gas), the XR group (irradiated), and the XR+H_2_ group (irradiated and hydrogen gas). From day 1 to day 5 of the experiment, the XR group was irradiated with 7 Gy each day. The XR+H_2_ group was placed in the hydrogen supply chamber and exposed to hydrogen for 2 h, then irradiated with 7 Gy, and finally exposed to hydrogen for another 2 h. From day 6 to day 14 of the experiment, the H_2_ group and the XR+H_2_ group were exposed to hydrogen for 2 h each day.

#### 2.3.6. Appearance of the Mice’s Dorsal Skin

The back skin of the mice in each group was observed and graded based on the modified Common Terminology Criteria for Adverse Events (CTCAE), as shown in Table 1 [23]. Skin appearance was assessed for each mouse on days 1, 7, 12, and 14 with accompanying photographs. The mice were sacrificed using CO_2_ on day 14 of the experiment, and the skin tissues were collected from the back with 2.0 cm × 2.0 cm in size.

#### 2.3.7. Histopathological Analysis

The skin tissue was fixed in 4% paraformaldehyde for at least 24 h at room temperature and then embedded in paraffin. Sections of paraffin-embedded skin were sliced and stained with hematoxylin and eosin (H&E) staining, Masson’s trichrome (MT) staining, and terminal deoxynucleotidyl transferase dUTP nick end labeling (TUNEL). The images were observed using a whole-slide scanning system (Zeiss Axioscan 7, Oberkochen, Germany).

#### 2.3.8. Preparation and Extraction of Protein Samples from Skin Tissue

Frozen skin tissues were homogenized and sonicated in either RIPA buffer (for Western blot analysis) or PBS buffer (for ELISA analysis) with protease inhibitor Cocktail I (EDTA-Free, 100× in DMSO, TargetMol, Boston, MA, USA) for 5 min, with the process repeated three times at 8500 rpm. Then, they were centrifuged at 13,200 rpm for 20 min at 4 °C. After obtaining the supernatant from the homogenates, the protein samples were stored at −80 °C.

#### 2.3.9. Western Blot Analysis

Protein concentration was determined using the Bradford Reagent (Sigma-Aldrich, St. Louis, MI, USA), and 30 µg of protein was used to prepare samples for electrophoresis. Equal amounts of protein were isolated in a 12% sodium dodecyl sulfate polyacrylamide gel. The proteins on the gel were then transferred to a polyvinylidene fluoride membrane, and the membrane was blocked with Blocking Buffer for 1 h. After blocking, the membranes were incubated with primary antibodies at 4 °C overnight. The primary antibodies used were β-actin at a ratio of 1:10,000 (Cat. No. GTX109639, GeneTex, Boston, MA, USA), GAPDH at a ratio of 1:5000 (Cat. No. GTX100118, GeneTex, San Antonio, TX, USA), Bcl-2-associated X protein (Bax) at a ratio of 1:3000 (Cat. No. GTX109683, GeneTex, TX, USA), B-cell lymphoma 2 (Bcl-2) at a ratio of 1:1000 (Cat. No. #3498, Cell Signaling, Danvers, MA, USA), IL-1β at a ratio of 1:1000 (Cat. No. A20529, ABclonal, Boston, MA, USA), and IL-6 at a ratio of 1:1000 (Cat. No. A0286, ABclonal, Boston, MA, USA). Subsequently, the membranes were washed three times with TBST buffer and incubated with secondary antibodies (1:5000, Goat Anti-Rabbit IgG antibody HRP, Cat. No. GTX213110-01, GeneTex, TX, USA) at room temperature for 1 h. The ECL reagent (Cat. No. GTX400006, GeneTex, TX, USA) was employed to detect the labeled proteins, and the images were captured using a Luminescence/Fluorescence Image System (GE Amersham Imager 680, Logan, UT, USA).

#### 2.3.10. Antioxidant Enzyme Analysis

The protein samples were quantified using the Bradford method before analysis. Superoxide dismutase (SOD) activity, catalase (CAT) activity, and glutathione (GSH) content in the skin were determined using specific assay kits from Cayman (SOD: Cat. No. 706002, CAT: Cat. No. 707002, GSH: Cat. No. 703002, Ann Arbor, MI, USA). For SOD activity, samples were diluted, added to a 96-well plate with Radical Detector and Xanthine Oxidase, incubated for 30 min at room temperature, and then absorbance was measured at 450 nm. For CAT activity, hydrogen peroxide was added to the diluted samples to initiate the reaction, which proceeded for 20 min before being terminated with potassium hydroxide; absorbance was measured at 540 nm. For GSH content, samples were mixed with metaphosphoric acid, centrifuged, and then the pH was adjusted with triethanolamine. After adding the detection reagent, the reaction was allowed to proceed for 25 min at room temperature before absorbance was measured at 410 nm. Standard curves and protein concentrations were used to calculate the SOD and CAT activities, as well as GSH content.

### 2.4. Statistical Analysis

Prism software (Version 8, GraphPad Software, Inc., San Diego, CA, USA) was employed for data analysis. Both in vitro and in vivo experiments were independently conducted at least three times (*n* ≥ 3 biological replicates). Results are presented as mean values with standard deviation (SD) represented by error bars. Group differences were analyzed using ANOVA followed by multiple comparison tests, with statistical significance defined as a *p*-value of less than 0.05.

## 3. Results

### 3.1. Protective Effects of Hydrogen Against Radiation Dermatitis In Vitro

We utilized HaCaT cells, immortalized human keratinocytes, to establish an in vitro model. HaCaT cells were incubated with different treatments: Ctrl, H_2_ (exposed to hydrogen gas for 1 h), XR (irradiation), and XR+H_2_ (exposed to hydrogen gas for 1 h, irradiation). The flow rate of hydrogen gas was 1.2 L/min. X-ray irradiation was conducted at 50 kV, with a total dose of 4 Gy. The selection of a 4 Gy dose for the cell experiments was based on previous studies and was also aligned with the dose per fraction typically used in clinical breast cancer radiation therapy [24,25,26]. The positive control was treated with a total dose of 10 Gy X-ray irradiation [27].

**Figure 1:**
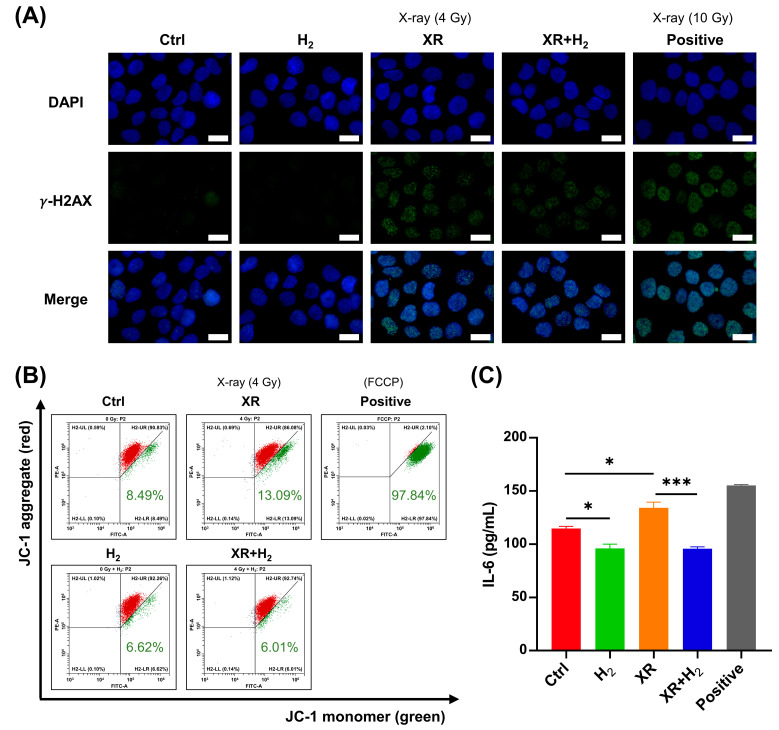
Effect of hydrogen gas on HaCaT cells in in vitro test. HaCaT cells were treated with or without hydrogen (H_2_) gas, followed by X-ray exposure (4 Gy). (**A**) Double-strand breaks were evaluated by staining nuclei (blue) with 4′,6-diamidino-2-phenylindole (DAPI) and γ-H2AX (green) with DyLight 488. Images were taken at 100× magnification, with a scale bar representing 20 µm. Cells exposed to X-ray irradiation (10 Gy) were used as the positive control. (**B**) Mitochondrial depolarization was analyzed by flow cytometry using JC-1 dye. Carbonyl cyanide-p-trifluoromethoxyphenylhydrazone (FCCP), a mitochondrial uncoupler that induces complete depolarization, was used as the positive control. Healthy mitochondria emitted red fluorescence (JC-1 aggregates, detected in the PE channel), while depolarized mitochondria emitted green fluorescence (JC-1 monomers, detected in the FITC channel). (**C**) Interleukin 6 (IL-6) levels were detected by an ELISA assay kit. Cells exposed to X-ray irradiation (10 Gy) were used as the positive control. Data are shown as the mean ± SD from three independent biological samples (*n* = 3). Statistical significance was determined using one-way ANOVA, with significance levels set at * *p* < 0.05 and *** *p* < 0.001.

Radiation-generated electrons and free radicals can induce DNA double-strand breaks, leading to cellular damage and death. The double-strand breaks prompt rapid phosphorylation of H2AX, resulting in the production of γ-H2AX [28]. As shown in Figure 1A, compared to the XR group, the XR+H_2_ group treated with hydrogen displayed fewer green fluorescent spots representing γ-H2AX, indicating the potential of hydrogen to reduce DNA breakage caused by radiation.

Ionizing radiation generates ROS, which may induce the opening of mitochondrial permeability transition pores, leading to mitochondrial depolarization (reduction in membrane potential) and subsequent cell apoptosis [29]. We utilized JC-1 dye to analyze MMP. In healthy mitochondria, the dye forms JC-1 aggregates emitting red fluorescence (PE-A channel) within the mitochondrial membrane. However, in apoptotic mitochondria, the dye forms JC-1 monomers, emitting green fluorescence in the cytoplasm [30]. In addition, FCCP, a mitochondrial uncoupler, was used as a positive control to disrupt the membrane potential, causing complete depolarization and green fluorescence in JC-1-stained cells. Figure 1B displays the results of MMP analysis conducted via flow cytometry. In the XR group, the percentage of green fluorescence is 13.9%, whereas in the XR+H_2_ group, it is only 6.01%. From this result, it can be inferred that hydrogen treatment can reduce radiation-induced cellular apoptosis.

To confirm whether hydrogen can effectively reduce radiation-induced inflammation, we measured the release of pro-inflammatory cytokines from HaCaT cells. Figure 1C displays the interleukin 6 (IL-6) levels released by HaCaT cells in each group. The IL-6 levels in the hydrogen-treated groups (H_2_ and XR+H_2_) are significantly lower than those in the Ctrl and XR groups, indicating that hydrogen reduces the release of IL-6 from HaCaT cells. In this study, IL-6 was analyzed 24 h after irradiation as an overall inflammatory marker, reflecting the immune response to radiation exposure and subsequent inflammation. Normalization of IL-6 secretion to cell viability was not performed, as the focus was on measuring the total inflammatory response.

### 3.2. Transdermal Ability of Hydrogen

**Figure 2:**
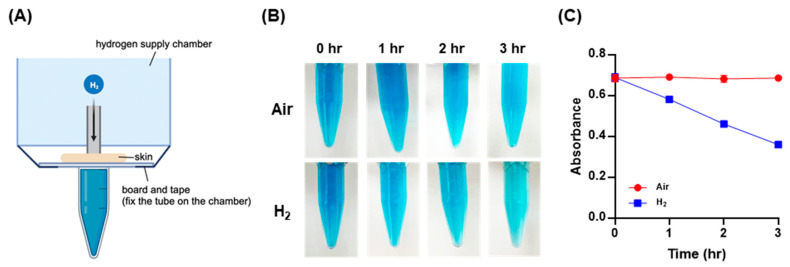
Hydrogen transdermal test. (**A**) Diagram of the transdermal test. (**B**) A photograph showing that the blue color of the MB-Pt probe disappeared after being reduced by H_2_ at different time points. (**C**) The absorbance at 662 nm of the MB-Pt probe after it was reduced by H_2_ (*n* = 3).

To verify hydrogen’s ability to penetrate the skin, we performed a transdermal test using the MB-Pt probe [31]. This probe transitions from blue to transparent (colorless) when exposed to hydrogen. The experiment entailed placing the probe in an Eppendorf tube with freshly harvested full-thickness mouse skin affixed on top as shown in Figure 2A. A hydrogen gas flow of 1.2 L/min was directed over the skin, and we observed a color change in the tube. Figure 2B illustrates the color change of the reagent from 0 to 3 h after exposure to hydrogen. Figure 2C presents the absorbance values of the probe at 662 nm measured at 0, 1, 2, and 3 h post-exposure to hydrogen. It is evident that the absorbance values of the probe placed in the air remained relatively constant, whereas those of the probe exposed to hydrogen continued to decrease over time. This suggests that the method of delivering hydrogen gas enables successful penetration of hydrogen through the skin.

### 3.3. Protective Effects of Hydrogen Against Radiation Dermatitis In Vivo

To assess the protective effects of hydrogen on RD, we conducted in vivo tests using BALB/c mice. The mice were randomly divided into four groups: Ctrl, H_2_, XR, and XR+H_2_. The Ctrl group comprised normal mice without radiation exposure or hydrogen treatment; the H_2_ group received hydrogen treatment only, without radiation exposure; the XR group received radiation exposure only; and the XR+H_2_ group received both radiation exposure and hydrogen treatment. The flow rate of H_2_ was 1.2 L/min for 2 h. X-ray irradiation was conducted at 50 kV, with a total dose of 35 Gy (7 Gy/day of fraction dose).

#### 3.3.1. Appearance of the Mouse’s Dorsal Skin

**Figure 3:**
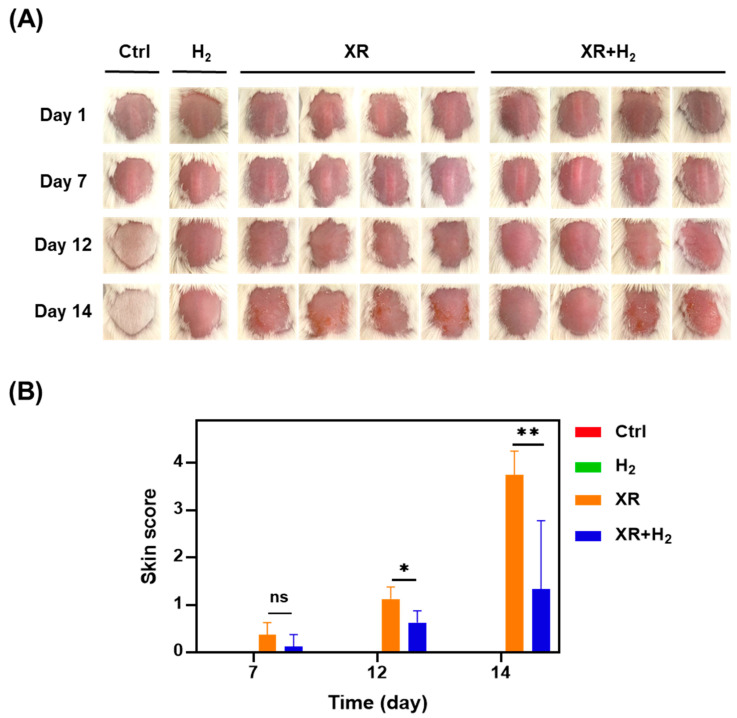
Appearance of the mice’s dorsal skin. (**A**) Photographs of the mice’s back skin were taken to investigate the in vivo radioprotection efficacy of H_2_ for radiation dermatitis. (**B**) Radiation dermatitis skin score of mice on day 7, day 12 and day 14. Statistical significance was determined using two-way ANOVA, with significance levels set at * *p* < 0.05 and ** *p* < 0.01. The ‘ns’ label indicates no statistically significant differences, with *p* > 0.05.

In Figure 3A, on day 7, no significant changes were observed in the skin of the radiation-exposed groups. By day 12, all mice in the XR group showed dryness and slight desquamation, while two mice in the XR+H_2_ group exhibited dry desquamation. By day 14, all mice in the XR group exhibited moist desquamation and extensive scabbing. In contrast, only two mice in the XR+H_2_ group showed scabbing, while the other two displayed no visible peeling or skin surface damage. Using the modified CTCAE scoring system (Table 1), we observed a significantly higher grade of RD in the XR group compared to the XR+H_2_ group on both day 12 and day 14 (Figure 3B).

#### 3.3.2. Anti-Inflammation Effect of Hydrogen on Radiation Dermatitis

**Figure 4:**
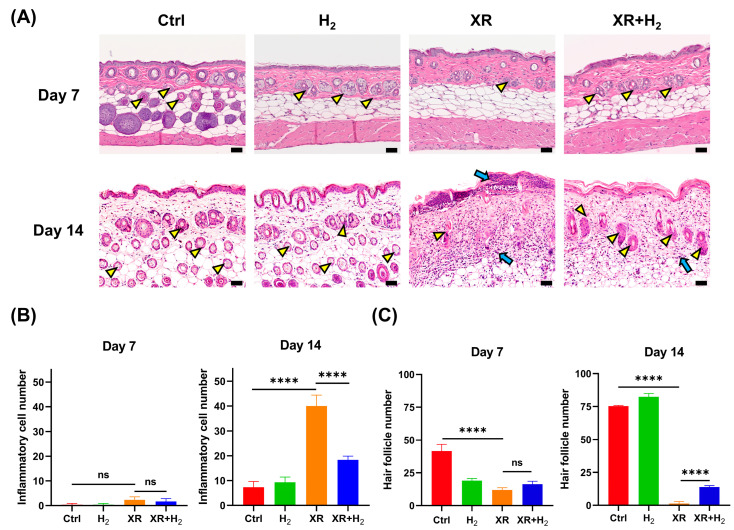
Hematoxylin and eosin (H&E) staining photomicrographs. (**A**) H&E staining, (**B**) inflammatory cell number per field of view, and (**C**) hair follicle number per field of view on the back skin of BALB/c mice on day 7 and day 14. The scale bar is 50 µm. The blue arrows indicate the location of inflammatory cells and the yellow arrows indicate the hair follicles. Data are shown as the mean ± SD from at least three independent biological samples (*n* ≥ 3). Statistical significance was determined using one-way ANOVA, with significance levels set at **** *p* < 0.0001. The ‘ns’ label indicates no statistically significant differences, with *p* > 0.05.

On day 7 and day 14, we conducted tissue biopsies to assess the skin response of mice following irradiation and hydrogen exposure. Figure 4 represents the H&E stain. By day 7, no notable differences were observed in skin histology between the XR and XR+H_2_ groups, with no inflammatory cell infiltration. By day 14, the XR group displayed significant epidermal disruption with scabbing and extensive leukocyte infiltration in the epidermis and dermis, indicating severe inflammation. In contrast, the XR+H_2_ group showed a relatively intact epidermis, fewer dermal inflammatory cells, and more surviving hair follicles.

**Figure 5:**
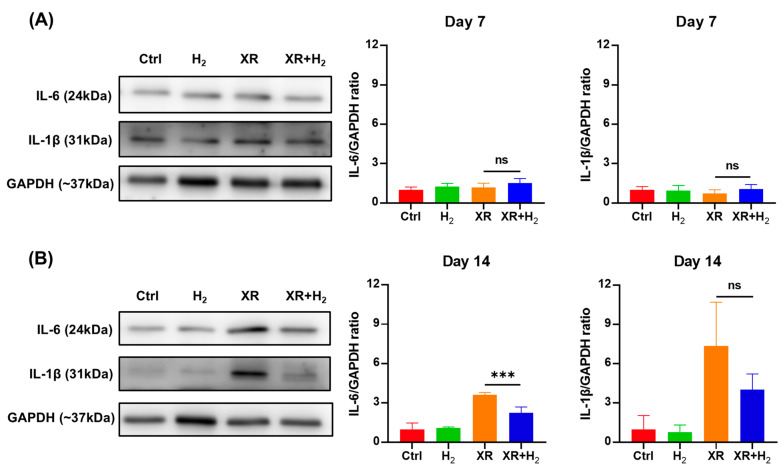
Regulation of the inflammatory markers by H_2_. Western blot analysis of IL-6 and IL-1β expression of mice skin on (**A**) day 7 and (**B**) day 14 of the experiment. Data are shown as the mean ± SD from at least three independent biological samples (*n* ≥ 3). Statistical significance was determined using one-way ANOVA, with significance levels set at *** *p* < 0.001. The ‘ns’ label indicates no statistically significant differences, with *p* > 0.05.

Radiation triggers immune responses, prompting the release of pro-inflammatory cytokines that induce inflammation. Figure 5 depicts the protein expression levels of IL-6 and IL-1β in the skin of mice in each group. On day 7, there were no significant differences in the expression levels of IL-6 and IL-1β among the groups. However, by day 14, a significantly lower expression of IL-6 and a decreasing trend in IL-1β were observed in the XR+H_2_ group compared to the XR group. Local administration of hydrogen could reduce the expression of inflammatory factors in irradiated skin, thereby mitigating radiation-induced damage.

**Figure 6:**
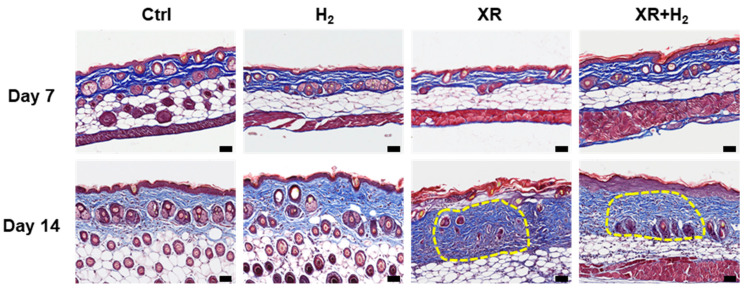
Masson’s trichrome (MT) staining photomicrographs. The top red region represents the epidermal layer and the blue-purple region represents the collagen fibers present in the dermal layer (yellow dashed line). The scale bar is 50 µm.

After exposure to radiation, the skin can produce an excess of ECM proteins, consisting of collagen, fibronectin, and proteoglycan [6]. We observed the content of collagen fibers in the dermis using MT staining. In Figure 6, the red color at the surface represents the epidermal layer, while the blue-purple color represents the collagen fibers present in the dermal layer. By day 14, the XR group shows a notable increase in collagen fiber accumulation in the dermis. Conversely, in the XR+H_2_ group receiving hydrogen therapy, there is a relatively lower collagen fiber amount, suggesting hydrogen’s potential to reduce ECM accumulation induced by radiation, thereby decreasing tissue hardness and thickness.

#### 3.3.3. Anti-Apoptosis Effect of Hydrogen on Radiation Dermatitis

**Figure 7:**
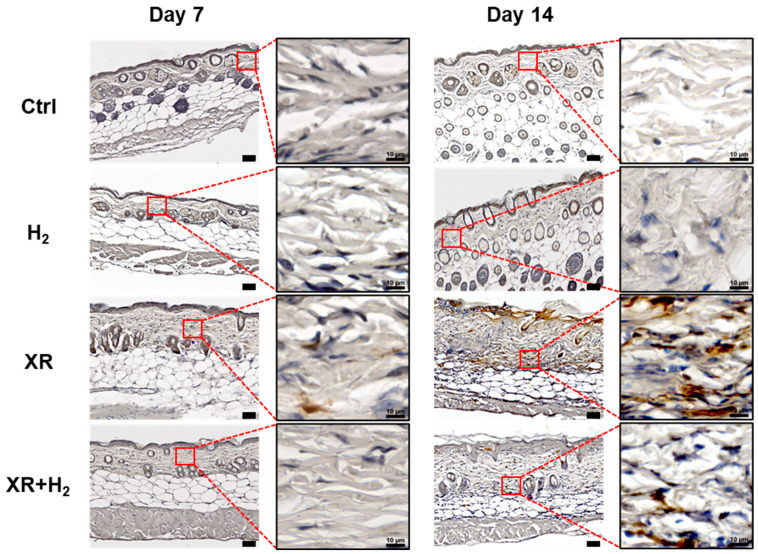
Terminal deoxynucleotidyl transferase dUTP nick end labeling (TUNEL) staining photomicrographs. The brown color indicates the location of apoptotic cells. On day 14, the XR+H_2_ group has fewer apoptosis signals than the XR group. Scale bars: 50 µm (left) and 10 µm (right) for each group.

TUNEL staining detects the location of apoptotic cells in tissues by labeling the DNA fragments. In Figure 7, the brown color represents the apoptotic sites labeled by TdT in the tissue. It can be observed that in the dermal layer of the skin tissue on day 7, only the XR group shows a few apoptotic cells. By day 14, the number of apoptotic cells in the XR group increases not only in the dermis but also in the epidermis and hair follicles, while the XR+H_2_ group exhibits fewer apoptotic signals compared to the XR group.

**Figure 8:**
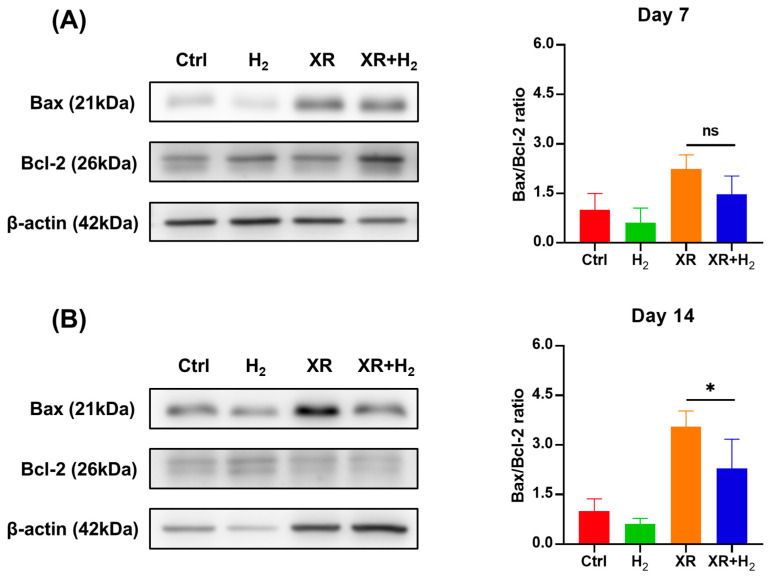
Regulation of the apoptosis-related markers by H_2_. Bcl-2-associated X protein (Bax) and B-cell lymphoma 2 (Bcl-2) expression on (**A**) day 7 and (**B**) day 14. Data are shown as the mean ± SD from at least three independent biological samples (*n* ≥ 3). Statistical significance was determined using one-way ANOVA, with significance levels set at * *p* < 0.05. The ‘ns’ label indicates no statistically significant differences, with *p* > 0.05.

Additionally, we examined the expression of apoptosis-related proteins in the skin of each group of mice through Western blot analysis. Bax is a pro-apoptotic factor, while Bcl-2 is an anti-apoptotic factor. The ratio of Bax to Bcl-2 protein expression determines survival or death after an apoptotic stimulus [32]. In Figure 8, the Bax/Bcl-2 ratios in mouse skin are depicted on day 7 and day 14, respectively. Compared to the Ctrl group, the H_2_ group showed a lower Bax/Bcl-2 ratio, indicating that hydrogen can reduce apoptosis in normal tissues. In the irradiated groups on day 14, there was a significant reduction in the Bax/Bcl-2 ratio in the XR+H_2_ group compared to the XR group, suggesting that local administration of hydrogen can effectively reduce radiation-induced apoptosis in skin tissue.

#### 3.3.4. Antioxidant Effect of Hydrogen on Radiation Dermatitis

**Figure 9:**
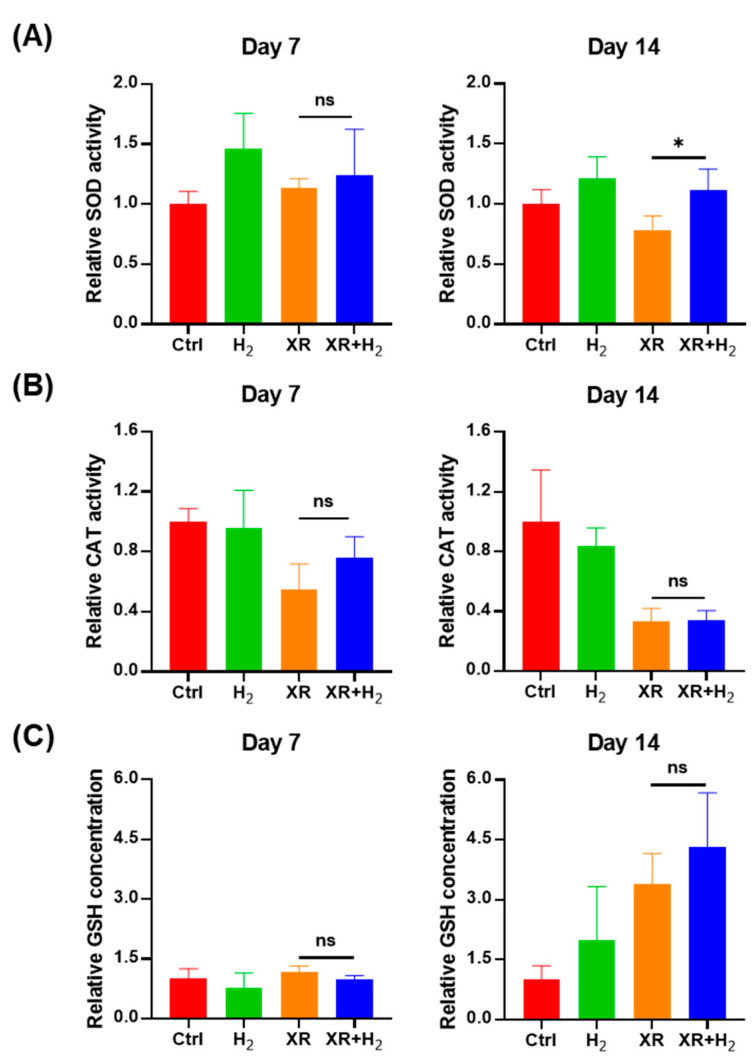
The effect of H_2_ on antioxidative enzymes and molecules. The activity and concentration of (**A**) SOD, (**B**) CAT and (**C**) GSH of mice’s skin on day 7 and day 14. Data are shown as the mean ± SD from at least three independent biological samples (*n* ≥ 3). Statistical significance was determined using one-way ANOVA, with significance levels set at * *p* < 0.05. The ‘ns’ label indicates no statistically significant differences, with *p* > 0.05.

Ionizing radiation generates an excess of ROS and induces oxidative stress, leading to inflammation and cell apoptosis. Therefore, we also aim to determine whether the protective effect of hydrogen on RD is related to the activity of antioxidant enzymes in the body. SOD is the most potent antioxidant enzyme in cells and plays a crucial role in combating ROS. It catalyzes the conversion of superoxide anion radicals into hydrogen peroxide and oxygen, thus preventing cellular damage [33]. In Figure 9A, the relative SOD activity in mouse skin on day 7 shows similar levels between the Ctrl and XR groups, with a slight increase noted in the H_2_ and XR+H_2_ groups, although not significantly different. By day 14, the XR group exhibits a decrease in SOD activity compared to the Ctrl group. Conversely, the XR+H_2_ group shows significantly higher SOD activity compared to the XR group, suggesting that local hydrogen administration protects SOD activity, thereby enhancing the skin’s capacity to eliminate superoxide anions.

The other two first-line defense antioxidants are CAT and GSH. Their primary function is to catalyze the reduction of hydrogen peroxide (H_2_O_2_) into water and molecular oxygen, thus completing the detoxification process initiated by SOD [33]. In our study, the use of hydrogen did not cause significant differences in the activity of antioxidant enzymes. However, we observed a trend where hydrogen could increase CAT activity in the early stages, but this activity decreased to the same level as the XR group later. On the other hand, continuous hydrogen treatment led to an increase in GSH levels.

## 4. Discussion

**Scheme 2:**
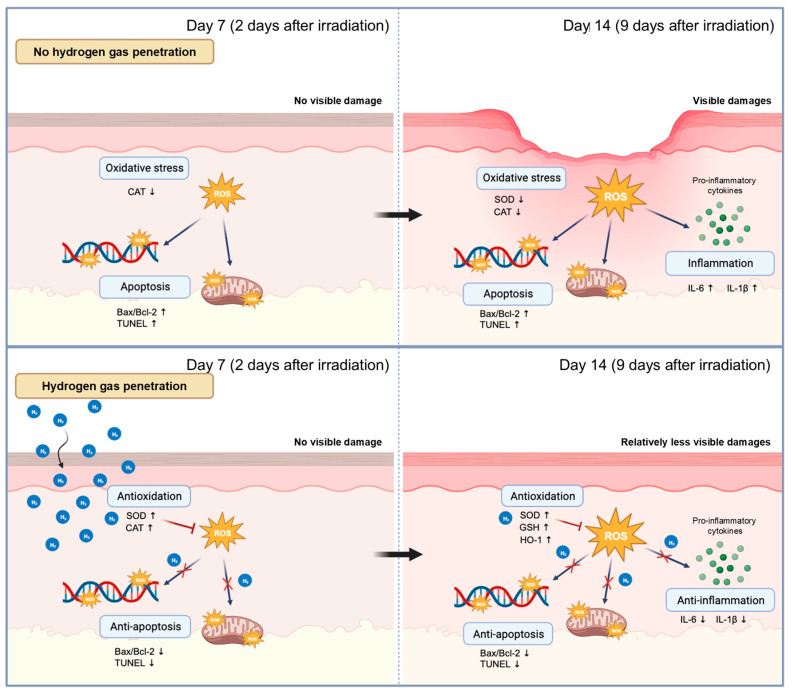
The possible mechanism of local hydrogen therapy in alleviating radiation dermatitis.

In this study, we have demonstrated the effectiveness of local hydrogen therapy in alleviating RD. The possible mechanism is demonstrated in Scheme 2. Hydrogen gas prevents RD by protecting skin cells from DNA and mitochondrial damage triggered by radiation exposure. It enhances antioxidant enzyme activity, thus mitigating the harm caused by reactive ROS and the subsequent release of pro-inflammatory cytokines, ultimately reducing the occurrence of apoptosis.

Several studies have explored the potential of hydrogen in managing RD. Mei et al. investigated the protective effects on RD of subcutaneously injected hydrogen solution [20]. This study primarily assessed efficacy through in vivo animal experiments, focusing on skin appearance and histological staining. Watanabe et al. utilized the inhalation of hydrogen gas as a treatment approach, demonstrating both preventive and therapeutic effects on RD [21]. Their in vivo experiments centered on evaluating skin appearance and conducting histological staining analyses related to apoptosis. Zou et al. explored the healing properties of hydrogen-rich water on RD through in vivo experiments, assessing changes in skin appearance and associated biomarkers following the onset of RT-induced wounds [22]. In our study, we opted to utilize pure hydrogen gas as the delivery method and explored its prophylactic effects through a combination of in vitro experiments using HaCaT cells and in vivo experiments involving BALB/c mice. Our investigation encompassed the evaluation of various parameters, including skin appearance, histology, and the expression of related proteins. Subjects were categorized into four groups: Ctrl (untreated), H_2_ (treated with hydrogen gas), XR (irradiated), and XR+H_2_ (treated with hydrogen gas and irradiated).

The in vitro cell experiments confirmed that hydrogen can reduce DNA damage and mitochondrial membrane depolarization in HaCaT cells caused by radiation (Figure 1). Hydrogen’s extremely low molecular weight enables rapid penetration into mitochondria and cell nuclei. It selectively scavenges harmful free radicals like hydroxyl radicals (•OH) and peroxynitrite anions (ONOO−), protecting HaCaT cells from radiation damage [16]. Additionally, administering hydrogen gas reduced membrane potential depolarization in non-irradiated cells. This effect may originate from hydrogen’s capacity to inhibit electron leakage in the electron transport chain, thus preventing its reaction with oxygen to generate ROS [34].

In in vivo experiments, through the assessment of skin appearance grade, H&E staining, and MT staining, it was observed that direct transdermal application of hydrogen can prevent radiation-induced follicle damage, dermal thickening, and leukocyte infiltration, thereby reducing the severity of RD. To further explore the role of hydrogen in protecting irradiated skin, we conducted an analysis of the expression of proteins associated with oxidation, inflammation, and apoptosis in the skin. The skin damage induced by RT progressively accumulates and becomes evident over time (Figure 3 and Figure 4). By day 7 (2 days after reaching a cumulative dose of 35 Gy), no notable disparity in skin appearance was observed between the XR group and the XR+H_2_ group. Furthermore, at day 7, there were no remarkable alterations in the pro-inflammatory cytokines IL-1β and IL-6 in the skin, which aligns with the absence of significant leukocyte infiltration observed in H&E staining (Figure 4 and Figure 5). Nevertheless, TUNEL staining unveiled a higher presence of apoptotic cells in the dermis of the XR group. Molecular biology assessments revealed a non-significant rise in SOD activity within the XR+H_2_ group compared to the XR group, indicating an augmented capacity to eliminate ROS, possibly correlated with the lower Bax/Bcl-2 ratio and reduced apoptotic cells in the XR+H_2_ group (Figure 7, Figure 8 and Figure 9).

By day 14 (9 days after the cumulative dose of 35 Gy radiation), the skin appearance and histology of the XR group exhibited significant damage (Figure 3 and Figure 4). This could be attributed to ROS produced by irradiation activating inflammatory responses, thereby stimulating the sustained production of additional nitric oxide (NO) and ROS through the iNOS or COX-2 pathway, leading to pathological changes in the skin [3]. In molecular biology experiments, the antioxidant enzyme SOD activity in the skin of the XR group mice was lower compared to the Ctrl group (Figure 9), suggesting that the excessive generation of ROS might lead to the consumption and inactivation of SOD [35]. This could subsequently result in cell apoptosis and inflammatory responses. The Bax/Bcl-2 ratio, indicative of apoptotic activity, was significantly higher in the XR group compared to Ctrl, along with noticeable apoptotic signals in the dermis observed through TUNEL staining and increased expression of pro-inflammatory cytokines IL-6 and IL-1β (Figure 5, Figure 7 and Figure 8). In the XR + H_2_ group, hydrogen treatment enhanced the skin’s antioxidant capacity, leading to a reduction in the Bax/Bcl-2 ratio, the number of apoptotic cells, and the expression of pro-inflammatory cytokines.

In addition to the antioxidant, anti-apoptotic, and anti-inflammatory effects mentioned above, hydrogen gas may prevent RD through exposure to high concentrations, which directly reduces the partial pressure of oxygen in tissue, thereby decreasing ROS generation and radiation damage. However, when choosing protectants for normal tissues during RT, ensuring they do not interfere with treatment efficacy is crucial, as the primary goal remains the therapeutic effect on the tumor. With oral or injectable administration routes, the agent is delivered to the target area via circulation, potentially resulting in a higher concentration in the tumor due to its rich blood supply. Consequently, utilizing these routes to prevent RD raises concerns. Although previous literature has suggested a potential synergistic effect between hydrogen and RT in tumor treatment, the exact mechanism remains unclear [16]. Therefore, our research design adopts local hydrogen gas exposure, where the hydrogen concentration in tissues depends on diffusion and tissue depth. This means that if the tumor is deep-seated, the impact of antioxidant therapy on tumor efficacy can be negligible.

We recognize several limitations in our study. Firstly, hydrogen exposure may alter pH and induce hypoxia, potentially inhibiting cell growth. Maintaining consistency between the experimental and control groups is essential in research. In this study, pH, temperature, and gas concentrations were not directly measured. Although the use of hydrogen/oxygen/CO_2_ mixtures was considered, concerns arose regarding the limited solubility of hydrogen in aqueous media, which might compromise the efficacy of hydrogen delivery in a mixed-gas system. Furthermore, the potential risks of combustion and explosion associated with mixed gases led to the selection of pure hydrogen for these experiments. No significant differences in DNA double-strand breaks, mitochondrial depolarization, or IL-6 levels were observed between the control and H_2_ groups, suggesting that the applied level of hydrogen exposure did not adversely impact cell growth. Further studies are warranted to determine whether prolonged hydrogen exposure may result in negative effects. Secondly, our choice of day 7 and day 14 as the evaluation time points in the in vivo experiment may not capture the dynamic and continuous nature of cellular responses. Moreover, the levels of antioxidant enzymes and cytokines in the tissue may not manifest the most significant differences at the time points we selected. Furthermore, by terminating the in vivo experiment and sacrificing the mice on day 14, we missed observing the recovery phase of RD. While our findings suggest that hydrogen gas may prevent RD, its potential therapeutic effects or ability to expedite recovery remain unknown.

## 5. Conclusions

In summary, our in vitro experiments highlight hydrogen gas’s potential to protect cells from radiation damage. Furthermore, the in vivo experiments reveal that exposing irradiated skin areas to a hydrogen gas environment reduces radiation-induced skin damages. This suggests that direct transdermal administration of hydrogen gas can effectively alleviate RD. In the future, transdermal therapy with hydrogen gas may be enhanced through the use of hydrogen-generating devices to facilitate administration.

## Figures and Tables

**Table 1:** Scoring system for radiation dermatitis (adapted from the Common Terminology Criteria for Adverse Events).

Score	Skin Appearance
0.5	Dryness
1	Dry desquamation
2	Early moist desquamation in a small area
3	Moderate patchy, moist desquamation (30% of the area)
4	Moist desquamation in most of the area (50% of the area)
5	Skin necrosis and ulceration

## Data Availability

The data presented in this study are available in the article.

## Supplementary Materials

The following supporting information can be downloaded at: https://www.mdpi.com/article/10.3390/antiox13121475/s1, Figure S1. Experimental setup for X-ray irradiation; Figure S2. Experimental setup for combined gas anesthesia and hydrogen exposure; Figure S3. Design of the neck plate setup.
