# Supplementary material for: Therapeutic Potential of Hydrogen as a Radioprotective Agent for the Prevention of Radiation Dermatitis
Source: Antioxidants (Basel). 2024 Nov 29;13(12):1475. doi: 10.3390/antiox13121475 (PMC11673486; doi:10.3390/antiox13121475)
Supplement: Supplementary file 1 [file antioxidants-13-01475-s001.zip › antioxidants-3267778-supplementary.pdf]

antioxidants

MDPI

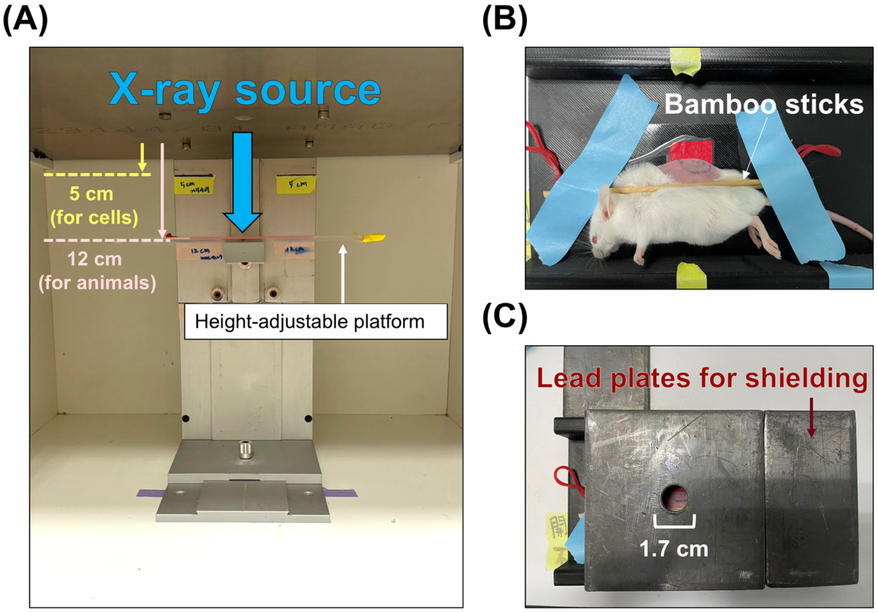

Figure S1. Experimental setup for X-ray irradiation. (A) The irradiation chamber with a height-adjustable platform. The platform can be positioned at 5 cm from the X-ray source for cell samples and 12 cm for animal experiments, ensuring appropriate dose administration. **(B)** Mouse fixation setup: mice were first anesthetized with ketamine and then securely held in place using bamboo sticks to prevent movement during irradiation. **(C)** Lead plates were used to shield non-targeted areas of the animal's body, exposing only the intended skin region to X-rays. This configuration minimizes unintended radiation exposure to other parts of the animal.

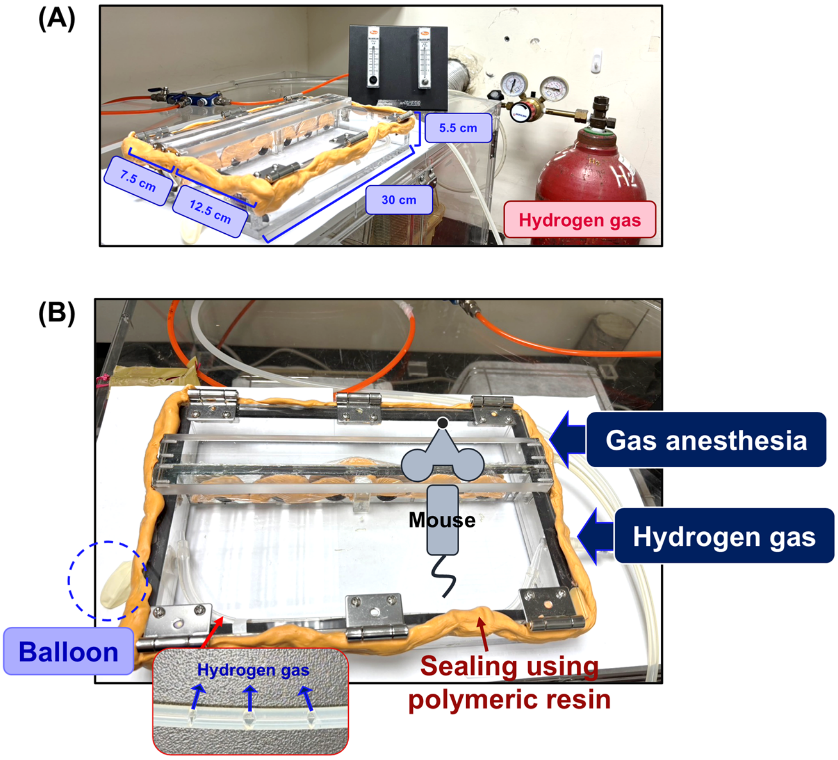

Figure S2. Experimental setup for combined gas anesthesia and hydrogen exposure. (A) Overview of the chamber dimensions and the flow of hydrogen gas from the tank into the sealed chamber.

Polymer foam materials and PE hydrogel are used to ensure secure sealing and prevent gas leakage. **(B)** Schematic illustration indicating the routes for gas anesthesia and hydrogen gas flow, with drilled tubing designed to ensure even distribution of hydrogen gas so that each mouse receives a similar amount of exposure. The balloon acts as a monitor for gas pressure, leak detection, and maintains slight positive pressure. The mouse illustration shows intended positioning; in practice, six mice will be placed in the chamber simultaneously. An inset provides a close-up view of hydrogen gas flow within the chamber. Polymeric resin sealing around the chamber edges ensures containment.

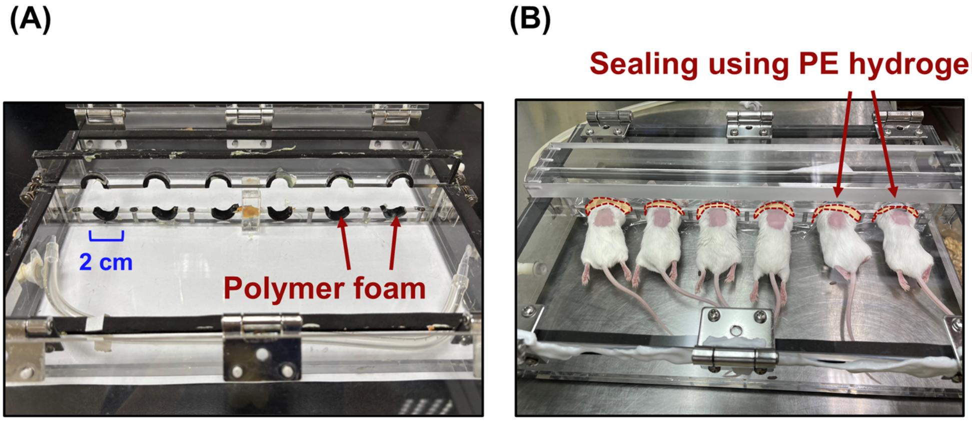

Figure S3. Design of the neck plate setup. (A) Interior view displaying foam materials positioned to maintain spacing, with a 2 cm scale bar for reference. **(B)** Arrangement of mice with neck plates sealed using polymeric resin to prevent substantial hydrogen leakage throughout the experimental procedure.
